# Autism and family involvement in the right to education in the EU: policy mapping in the Netherlands, Belgium and Germany

**DOI:** 10.1186/s13229-019-0297-x

**Published:** 2019-12-09

**Authors:** Robin van Kessel, Andres Roman-Urrestarazu, Amber Ruigrok, Rosemary Holt, Matt Commers, Rosa A. Hoekstra, Katarzyna Czabanowska, Carol Brayne, Simon Baron-Cohen

**Affiliations:** 1 Department of International Health, School CAPHRI Care and Public Health Research Institute, Faculty of Health Medicine and Life Sciences, Maastricht University, Minderbroedersberg 4-6, 6211 LK Maastricht, The Netherlands; 2 Institute of Public Health, University of Cambridge, Forvie Site, Robinson Way, Cambridge, CB20SR UK; 3 Autism Research Centre, Department of Psychiatry, University of Cambridge, 18B Trumpington Road, Douglas House, Cambridge, CB2 8AH UK; 4 Department of Psychology, Institute of Psychiatry, Psychology & Neuroscience, King’s College London, Addison House, Guy’s Campus, London, SE1 1UL UK; 5 Department of Health Policy Management, Institute of Public Health, Faculty of Health Care, Jagiellonian University, Krakow, Poland

## Abstract

**Introduction:**

In recent years, the universal right to education has been emphasised by the Universal Declaration on Human Rights and the Convention on the Rights of Persons with Disabilities. In this paper, we mapped policies relevant to special education needs and parental involvement of children with autism at an international level and in the Netherlands, Germany and Belgium.

**Methods:**

A policy path analysis was performed using a scoping review as an underlying methodological framework. This allowed for a rapid gathering of available data from which a timeline of adopted policies was derived.

**Results and discussion:**

Internationally, the universal right to education has been reinforced repeatedly and the values of the Universal Declaration of Human Rights have been reiterated with every reinforcement. Also, the additional support that a child with special education needs requires is acknowledged and measures are taken to facilitate access to any education for all children. There are slight cross-country differences between the countries under study, attributable to differences in national regulation of education. However, all countries have progressed to a state where the right to education for all children is integrated on a policy level and measures are taken to enable children with special needs to participate in education. Recently, an attempt to implement a form of inclusive education was made as a form of special needs provision. Nevertheless, nowhere has this been implemented successfully yet.

**Conclusion:**

The Universal Declaration of Human Rights was a critical juncture in international policy and created an environment where the universal right to education has been implemented for all children in the countries under study.

## Introduction

Autism spectrum conditions (ASCs, henceforth autism) are lifelong developmental conditions that cause difficulties in reciprocal social interaction and communication, alongside unusually repetitive behaviours and narrow interests, sensory hyper- or hypo-sensitivity and difficulties in adjusting to unexpected change [1]. Autism is present in 1% of the population with a male-to-female ratio between 3:1 and 4:1 [2, 3]. Autistic individuals may be more prone to serious health and other functional difficulties, which may lead to financial problems for families and carers, and can carry considerable stigma [4–7]. To address these issues and increase the quality of life and inclusion of people with autism and the autism community across Europe, the application of fundamental rights of education in the European Union (EU) is paramount [8].

Consequently, it is crucial for EU Member States to provide special education needs (SEN) services from early childhood onward, and throughout school years, while supporting people in life-long education to allow people with autism to achieve their full potential [2, 9]. Examples of SEN as defined by Carroll and colleagues [10] include linguistic difficulties, problems in communication, or learning disabilities that require additional support provision. Schools play a vital role in the provision of SEN services, such as providing support with language, planning, social interactions and communication. An example of SEN service provision in schools is inclusive education. For this paper, the term inclusion is best explained by Grindal and colleagues [11]: ‘a process of systemic reform embodying changes and modifications in content, teaching methods, approaches, structures and strategies in education to overcome barriers with a vision serving to provide all students of the relevant age range with an equitable and participatory learning experience and environment that best corresponds to their requirements and preferences.’ In other words, the necessary SEN services are provided in mainstream education (conventional education that neurotypical children also attend), along with other structural changes, thus enabling children with autism to participate with their peers [10, 11]. The vital role of schools is further stressed by the large amount of time children spend in an educational setting and, by extension, the potential that education has as an essential place to address the difficulties that autistic children experience [10, 11]. Providing these services appropriately and adequately can yield significant short- and long-term benefits for a child’s cognitive and social development [11]. Significant improvements in the development of social skills have been found in children with autism attending mainstream or mixed schools [12], with enhanced engagement with the environment [13], allowing for opportunities for improvement in independence and self-sufficiency [14]. Furthermore, children that receive proper SEN services are twice as likely to enrol in secondary post-education. For example, in an independent large-scale study in the USA [15], with 11% of children being more likely to be employed [16], and 10% being more likely to be able to live independently [12, 16]. Currently, approximately 9.3% of children receive SEN services in the Netherlands [17], and Belgium is split between 6.2% in Flanders and 4.5% in Wallonia [18]. The proportion in Germany is currently unknown, as it has not yet been investigated.

SEN services are implemented differently across countries [19]. In the Netherlands, there is a distinction between mainstream, mixed and special schools [20]. Within the Dutch system, there are schemes in place that can provide financial support to obtain additional care when attending a mainstream or mixed school. In the EU’s largest nation, Germany, the main focus is towards inclusion of all children into a single school system, while also providing special schools for those that find the provision of special needs in mainstream schools to be insufficient [21]. Local and regional dynamics play an important role as, for example, the SEN provision system in Belgium is shared between the regions of Flanders, Wallonia, and the German community [22]. Similarly, each Federal State (Land) in Germany is responsible for their own implementation of SEN services [23]. In Flanders, a clear separation between mainstream and special schools remains, even though new incentives to create room for mixed schools were introduced in 2014 [24]. Wallonia, on the other hand, created a step-wise system of inclusion into mainstream schooling that depends on the ability of a child and the severity of the condition [25].

Family support and engagement in both policymaking and education play a key role in supporting children with SEN and improving health outcomes [26]. For example, Northern Ireland was the first country to explicitly stress the importance of education and policy engagement of family members in their Autism Strategy (2013–2020). The Autism Strategy was aimed at involving parents in the education process of their child with autism [27]. In the National Plan for Autism in Children in the UK, active family involvement was one of the core policy principles in taking care of an child with autism [28]. It specifies that families and carers, in the form of parent-professional partnerships, are involved in the accomplishment of development goals set for the child with autism. The extent to which other Member States in the EU have implemented family involvement in their autism policy in SEN is currently unknown, although previous work by Roleska and Roman-Urrestarazu and colleagues has investigated EU SEN policy overall [29].

This paper is a continuation of the European Consortium for Autism Researchers in Education (EDUCAUS) project [30] and builds upon previous work that started mapping EU SEN policy in all 28 Member States [29, 31] by expanding the scope with three EU Member States to look specifically at family involvement in autism policy. Through a scoping review, we comparatively assess and map family involvement in EU and local policymaking in the Netherlands, Germany and Belgium. Both the Universal Declaration of Human Rights (UDHR) [32] and the Convention on the Rights of Persons with Disabilities (CRPD) [33] play a key role in the EU disability policy through their respective endorsement of inclusive education of people with autism and the fact that both documents have been signed by all EU Member States. Therefore, we employ these as underlying principles when conducting the policy path dependence analysis. In doing so, the main policy outcomes are shown, as well as to what extent these outcomes are connected between various levels of policymaking (e.g. international, national and regional). Ultimately, our goal is to establish a clear policy perspective on how families are integrated in policy that ensures the right to education is established, as well as how SEN interventions might affect public health outcomes for people with autism across three Western EU countries: the Netherlands (17.1 million people) [34], Germany (82.5 million people) [34], which is represented by Bavaria, North Rhein Westphalia, Saxony and Lower Saxony in this scoping review, and Belgium (11.3 million people) [34], which is divided in Flanders (6.4 million people) [35], Wallonia (3.6 million people) [35], the German-speaking region (75.2 thousand people) [35] and the Brussels-Capital region (1.1 million people) [35]. Since Germany is a federal republic, meaning every land is responsible for regulating education within its respective territory, Bavaria, North Rhein Westphalia and Lower Saxony were chosen since they are the largest Länder in terms of population [36]. Saxony, being a former Eastern German Land, was added as a comparison point to account for the former separation of Germany, since it has a population size similar to Lower Saxony. As a result, an adequate representation of the overall German environment is ensured. Further reasons for the selection of these countries are their proximity, similar income levels and standards of living, comparable health and social welfare systems, cultural likeness, their autism prevalence (the Netherlands, 0.6–2.3%, Germany 0.4%, Belgium 0.6%) [20, 37, 38], and their demographic weight. Together, they represent a considerable portion (21.6%) of the total EU population (511.5 million people) [34] that might be affected by autism-specific policies [39, 40]. A table showcasing all this data is included in an additional file (see Additional file 1). Ultimately, this study adds to the existing body of literature on autism treatment by providing a social policy perspective. It also adds to the epidemiological body of literature by highlighting differences in vulnerability across countries and, in particular, the need to focus on differential evidence bases for public health policies directed towards different levels of need across different regions.

## Methods

The study uses the policy mapping framework previously used and validated by Roleska and Roman-Urrestarazu and colleagues [29]. It is well suited for the scope of this research based on the implementation and development of public health policy. Also, a scoping review allows for the rapid mapping of the key concepts underpinning a broad research area that is particularly valuable for complex issues which have not been reviewed comprehensively to date [41, 42]. In other words, this methodology encompasses the structural and systematic nature that a systematic literature review would bring, while it disregards the quality assessment of data gathering due to the scarce body of existing literature. This scoping review and mapping exercise were conducted by means of a policy path dependence analysis [43]. This methodology is particularly suited for analysing the development of policy based on pre-existing legislation (e.g. the UDHR and CRPD) combined with contingent factors, such as external coercive pressure [43]. It also integrates competing ideas and values (e.g. international versus national priorities), which allows for the exploration of interactions among different countries as well as how they follow supranational guidance (e.g. United Nations or EU guidance). Because there is no single, representative data source in the EU with regards to autism and SEN policy, we adopted a modular approach to legislative and policy work across the different educational policy layers of analysis (International, EU and Dutch, German and Belgian-specific). We used the PRISMA framework to report our findings [44].

### Theoretical framework for data analysis and path dependency

An analysis of policy path interdependency was performed drawing on current and past international, EU and national policies in the field of education and autism. Path dependence technique enables the identification of policy-making patterns and establishes influences and interrelations among policies in linear layers of temporality [43]. It also allows for policy process-tracing, which firstly aims to clarify what factors are present in critical policy junctures. Secondly, it aims to create a reference framework and illustrate how decision processes come to conclusions. Thirdly, it aims at clarifying how behaviour that occurs in different stakeholders as a response to external factors (e.g. the adoption of new policy) affects various institutional arrangements (e.g. collaborations between institutions) [45, 46]. In this case, the UDHR was the initial policy (1948), a milestone document that influenced both the creation and the content of EU as well as national policies. Time and policy were two variables presented on a timeline to show their linkage and overlap to facilitate further analysis. This enabled us to see policy creation as historical sequences and patterns and identify path dependence [43]. Current disability and autism policies are a result of previous events that were tracked using this framework. Each policy was analysed by identifying its input in the field of education, pros and cons, as well as in relation to other policies.

### Eligibility criteria

Since this report expands the work on EU SEN policy mapping of the 28 EU Member States, the scope of policies was limited to autism and SEN policies that relate to the national education system, the right to education, special needs education and disability laws. Additionally, policies and documents relating to autism and educational policy of those younger than 18 years, with any comorbid health condition in any setting, were eligible for inclusion. Legal documents provided by governments were included, whereas programs and strategies developed by non-governmental organisations were excluded. Furthermore, legislation was eligible for inclusion as long as it was published after 1945. Constitutions were included regardless of publication date, because of their fundamental role in legislation.

### Data collection and search strategy

The first step in this policy mapping was to review and extract relevant policies and legislation that address the right to education of people with autism directly from original governmental sources. Several databases were used in the collection of data. The United Nations database (http://www.ohchr.org) was used for the retrieval of its policy documents. Eur-Lex (http://eur-lex.europa.eu) and N-Lex (http://eur-lex.europa.eu/n-lex/) were used to search for EU and national governmental documents respectively. Additionally, Kluwer Navigator (https://www.navigator.nl) was used for more detailed searches in Dutch legislation, JURIS (https://www.juris.de) was used to search for national and regional German legislation and BelgiëLex (http://belgielex.be) was used to access the Belgian legislation (divided in Flanders, Wallonia and the German community). No limits were put on language. Moreover, no time limit was used during the searches, as the goal was to create a timeline of policies. The second step was to develop a multi-faceted search strategy for electronic databases (PubMed and Google Scholar) that was executed by a single researcher. A selection of key terms was created to use as the foundation of the search terms: ‘autism; disability; SEN; education; law; policy; right to education; special needs; special education; inclusive education’. Next, the academic databases PubMed and Google Scholar were searched using the following combinations of search terms: ‘autism & disability’; ‘autism & SEN’; ‘autism & education’; ‘autism & law’; ‘autism & policy’; ‘SEN & disability’; ‘SEN & law’; ‘SEN & policy’; ‘disability & law’; ‘disability & policy’. The final search query is shown in Table 1, along with its constituent terms. The national policy depositories were searched using the separate key terms, as combining the search terms yielded little results. Adaption of the policy search strategy was done through the translation of the search terms in Dutch, German and French respectively. The third step consisted of merging policy and academic publications according to the eligibility criteria. The fourth step was acquiring further information through searching reference lists and grey literature (e.g. the website of the European Agency for Special Needs and Inclusive Education and the Eurydice Network [47, 48]). Policy documents and governmental strategies in the countries under study were compared to the EU disability and educational policy. In case documents were not present, general disability policies and legislation were analysed. The data collection was built on the appraisal of three searches: one looking for autism and educational policy internationally, one at the EU level and one at the national level. In the cases of Germany and Belgium, the sub-national level was analysed as well, considering their identity as federal states. The final step was unifying the three searches into one single data repository for the purpose of the scoping review. The final dataset was then verified by both lead authors to maximise screening reliability.

**Table 1:** The build-up of the final search query for academic databases

	Search query
Term 1	((((((((((autism & law) OR autism & policy) OR autism & SEN) OR autism & education) OR autism & disability) OR SEN & policy) OR SEN & law) OR disability & law) OR disability & policy))
Term 2	((Netherlands OR Belgium OR Germany))
Final query	((((((((((autism & law) OR autism & policy) OR autism & SEN) OR autism & education) OR autism & disability) OR SEN & policy) OR SEN & law) OR disability & law) OR disability & policy)) AND ((Netherlands OR Belgium OR Germany))

## Results

**Fig. 1:**
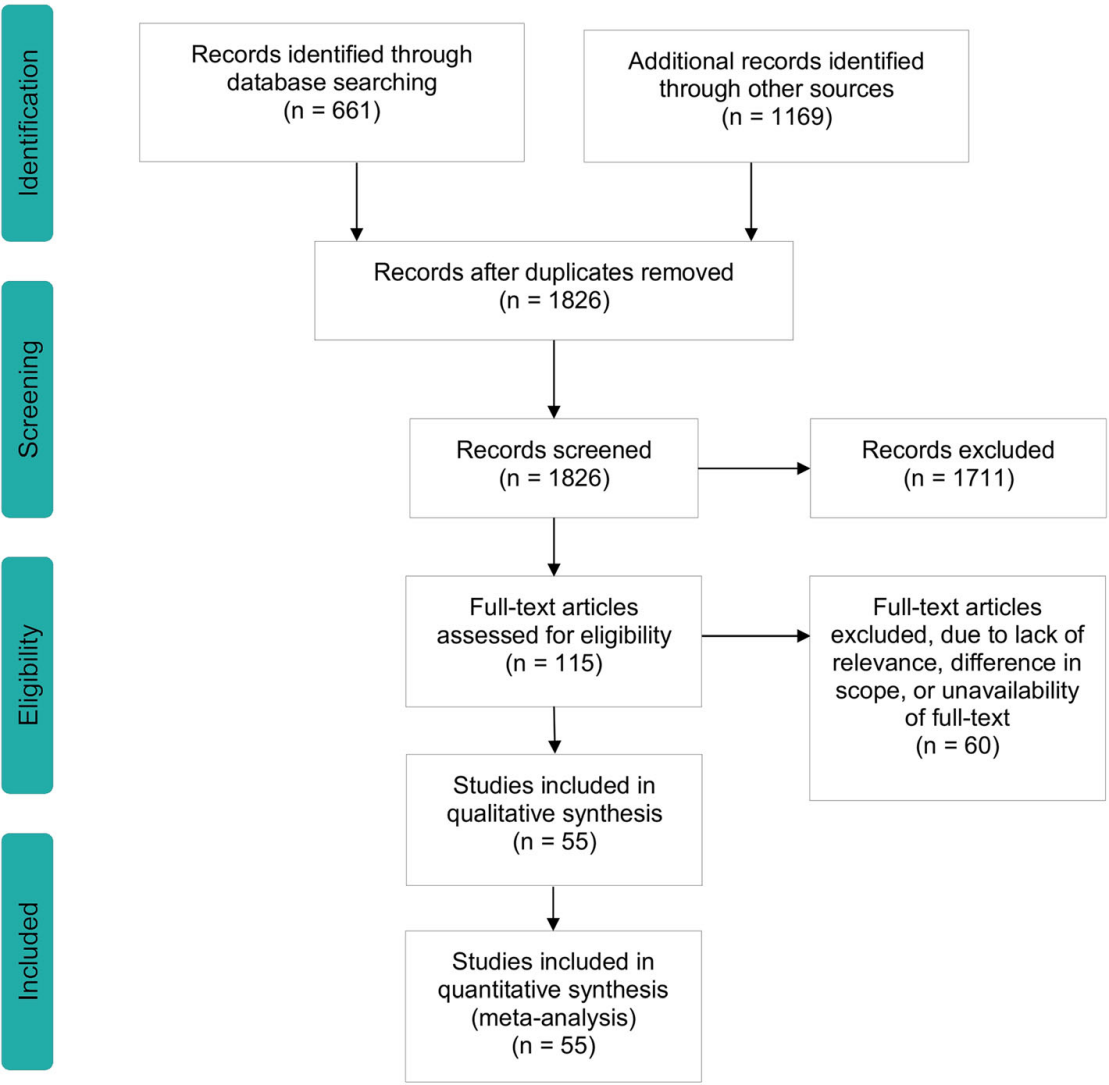
An overview of the data collection process

We identified 661 sources through academic database searching and 1169 through policy databases. A PRISMA flowchart illustrates this process in Fig. 1 where policy databases are referred to as ‘other sources’. Four duplicates were identified and removed, leaving 1826 sources to be analysed using the eligibility criteria. After analysing titles and abstracts, 115 sources were considered eligible for full-text screening. An example of an excluded item is the annual report on the overall spending of the Ministry of Education. It matches the search criteria (keyword ‘Education’), yet does not fall within the scope of this report, since it is not directly policy-related. The full-text screening resulted in the exclusion of another 60 articles, due to lack of relevance, difference in scope and unavailability of the full text. The remaining 55 articles were included in the scoping review. It has to be noted that the number of documents identified through policy searches far exceeds the number that was found using the academic databases. There are two explanations for this: (1) the two databases were searched using different search terms, as the search terms for the academic databases were not transferable to the policy databases and the single key terms had to be used to access the data; and (2) the current body of literature on SEN policy is still heavily in development.

**Fig. 2:**
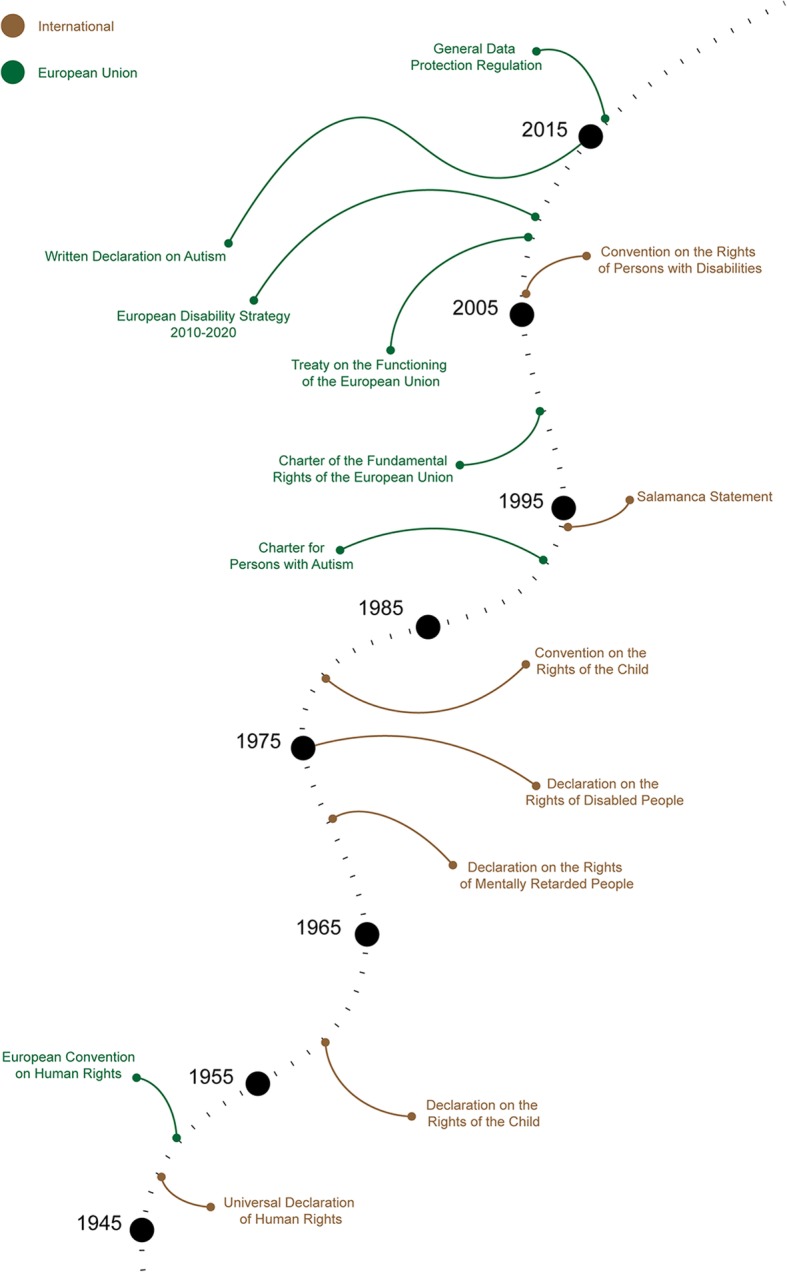
A chronological illustration of international and EU policies in the field of SEN

**Fig. 3:**
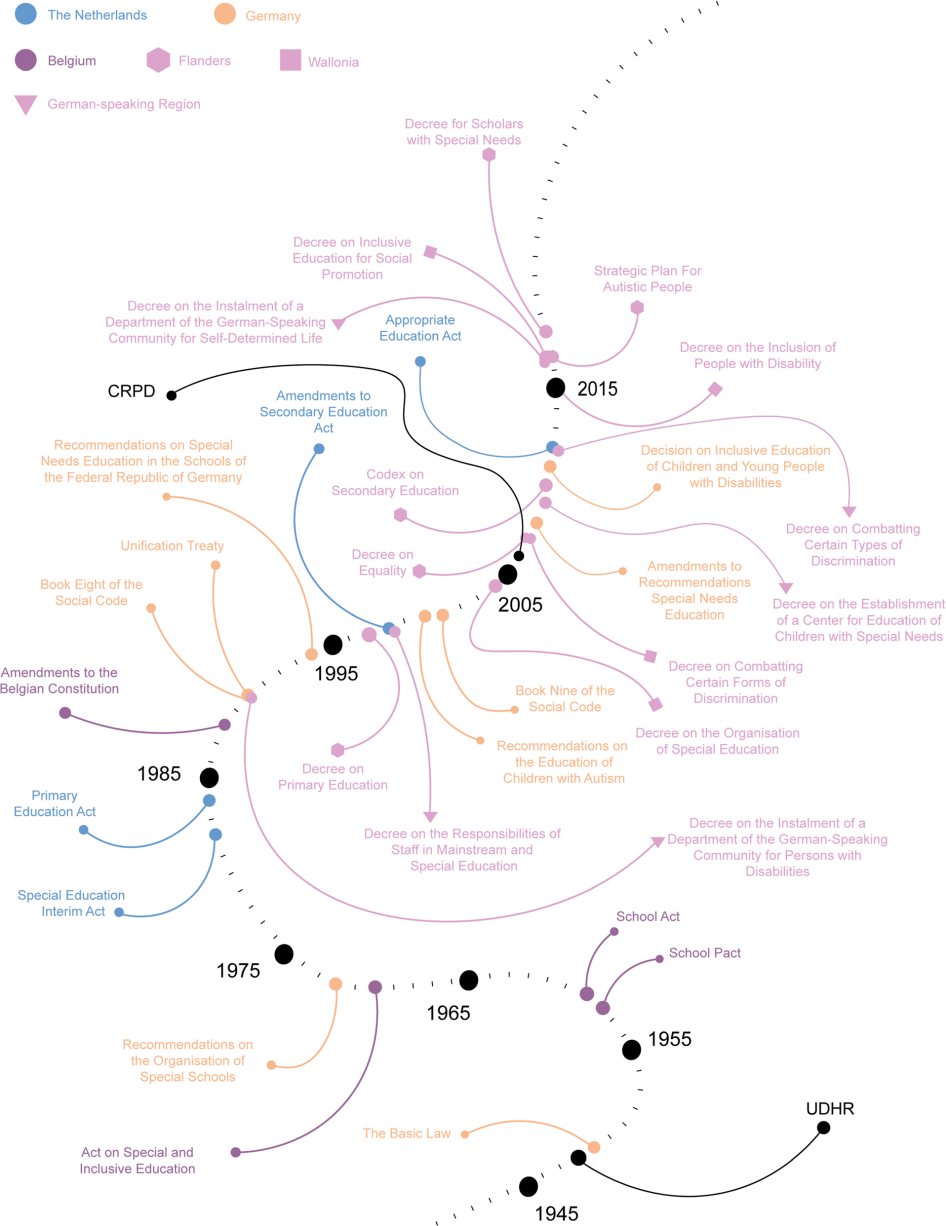
An illustration of all included national and subnational policies with regards to SEN

A timeline was constructed to illustrate the adopted policies relating to family involvement and right to education in autism, both internationally and nationally. Figure 2 illustrates the timeline of the international policy, while Fig. 3 captures the timelines of the individual countries. A concise overview of the findings is provided in two additional tables (see Additional file 2 and Additional file 3). The preeminent act that stresses the importance of right to education is the UDHR [32]. It stipulates in Article 26 that everyone is entitled to free education in the elementary and fundamental stages and that education should be aimed at fully developing the individual. This right is expanded in the Declaration on the Rights of the Child [49], where Article 24 states that special needs of children should be met when it comes to treatment, education and care. Finally, the CRPD emphasises that children with disabilities should be able to fully enjoy all human rights and fundamental freedoms on an equal basis with other children, thus including the right to education once more as one of its central tenets and therefore cementing any future development of children and people with disabilities and their right to education [33].

### International policy

The UDHR can be considered the critical juncture upon which the current SEN policy environment is based, since it is the first international document to specify the right to education for everyone, the right for each individual to fully develop his or her potential and the right of parents to be included in the educational process of their child. While the UDHR acknowledges that everyone has the right to education, the United Nations also acknowledges that children may require special care and support [49]. Consequently, the Declaration on the Rights of the Child was adopted in 1959. In the fifth principle of this Declaration, it is explicitly stated that a child who is physically, mentally or socially handicapped should be given appropriate treatment, education and care that matches the particular condition. After the adoption of these two legislative acts, children with special needs in all signatory countries were entitled to education without discrimination based upon, for example, their disability or autism status. While there was a solid foundation for general human rights at this point, the rights of people with disabilities were not specifically addressed. This changed with the adoption of the Declaration on the Rights of Mentally Retarded Persons in 1971 and the Declaration on the Rights of Disabled Persons in 1975 [50, 51]. Article 2 of the Declaration on the Rights of Mentally Retarded Persons stresses the right of people with intellectual, developmental and learning conditions specifically to receive appropriate education in order to develop themselves to their maximum potential [50]. In addition, Article 4 covers the necessity for people with neurodevelopmental conditions to live with their own family where possible, as well as the need for the families with whom they live to receive assistance. Nevertheless, the scope of this Declaration is limited to people with intellectual, developmental, or learning conditions only. The Declaration on the Rights of Disabled Persons was signed shortly after to expand this scope to people with disabilities in general [51]. The definition of ‘disabled person’ that was established in this Declaration allowed the inclusion of autism as a disability. The Convention on the Rights of the Child expands on the principles of the Declaration on the Rights of the Child and the rights and duties of the parents to provide direction to the child with regards to its development [52]. After its adoption in 1989, the role of the parents in the development of their child was closely explained from a human rights perspective. Previously, it had only been established that parents play a general role in the development of the child. Moreover, United Nations Educational, Scientific and Cultural Organisation (UNESCO) published the Salamanca Statement in 1994 [53], which reemphasises the fundamental right to education for every child. Moreover, the Statement acknowledges the unique characteristics and learning needs for every child and calls for education systems to be designed to take this wide diversity of characteristics and needs into account, making it so that children with SEN can follow mainstream education with additional supportive services.

### European Union

Shortly after the adoption of the UDHR by the United Nations, the Council of Europe adopted the European Convention on Human Rights in 1952. Its Protocol laid down the right to education for every person in what later came to be the European Union [54]. As a response to the shift of the international discussion on human rights to rights for persons with disabilities, the Charter for Persons with Autism was created in 1992 by Autism-Europe and was adopted as a Written Declaration by the European Parliament in 1996 [55]. It directly refers back to the Declarations on the Rights of Mentally Retarded Persons and the Rights of Disabled Persons. Furthermore, its scope is specifically aimed at people with autism, as it emphasises their rights to accessible and appropriate education (point 3) and the equipment, assistance and support services necessary to live a fully productive life with dignity and independence (point 6). When the Treaty of Amsterdam was signed in 1997, the aim to combat any form of discrimination was reiterated once again, while explicitly specifying discrimination on basis of disability (Article 6a) [56].

With the adoption of the Charter of Fundamental Rights of the European Union in 2000, all rights and freedoms that were previously protected by different legislative pieces were pooled into one legally binding document in an attempt to create a closer Union with common values (Preamble) [57]. The right to education for everyone is stated in Article 14, although disabilities and autism are not specifically mentioned in this document. However, it does state that the pedagogical convictions of the parents need to be respected with regards to the education of the child, as long as it remains in accordance with national laws. When applied to the case of SEN service provision, it would allow parents to adopt a decisive role when choosing the form of education, as long as it remains within the boundaries of national law. Additionally, the competence of education was delegated completely to the EU Member States after the ratification of the Treaty on the Functioning of the European Union (2009) [58]. It states that the role of the EU is to encourage cooperation and provide support where necessary (Article 165). This would allow every Member State to regulate its own education system and, by extension, its approach to SEN. However, in doing so, it creates a heterogeneous environment in the approach to SEN service provision, as education systems vary from country to country.

The European Disability Strategy 2010–2020 builds upon the Charter of Fundamental Rights of the European Union, the Treaty on the Functioning of the European Union and the Convention on the Rights of Persons with Disabilities. The Strategy aims to empower people with disabilities so that they can enjoy their full rights. It acknowledges that children with disabilities are often not included in mainstream education and calls for them to be integrated appropriately through the use of inclusive education, along with individual support [8]. Additionally, the Written Declaration on Autism was adopted by the European Parliament in 2015 [59]. It calls for an EU Strategy on autism specifically, as it recognises that early detection is still lacking across Europe, despite the importance of early diagnosis to provide appropriate and adequate support and education (point 3). Furthermore, it attempts to encourage research on autism, prevalence studies and exchange of best practices regarding evidence-based interventions for children with autism, as well as support services and services for adults that teach and improve their skills for addressing and coping with autism in daily life (point 5). Finally, with the introduction of the General Data Protection Regulation in 2018 [60], the data on health conditions is prohibited to be used in any way without the consent of the parents or guardians (Article 9). As a result, implementation of SEN services has become more complex when it involves multiple stakeholders, as health data cannot be shared as freely as before. Consequently, the institution that is responsible for the provision of SEN services is tasked with the collection of informed consent of the parents before the process of determining appropriate SEN services based on the condition of the child can be started.

All in all, EU legislation early on mainly focused on creating a binding basic human rights framework. Autism has also been acknowledged early on in the history of the EU as part of establishing rights for people with disabilities. However, further action on empowering people with autism did not happen until the EU Strategy in 2010 and the Written Declaration in 2015. Finally, the focus of EU legislation is put predominantly on the individual with disability, while the direct environment is mostly disregarded so far.

### The Netherlands

Compulsory education has been implemented for all children in the Netherlands since 1901 [61]. This education takes the form of mainstream schooling or home schooling. A framework for schools that focus specifically on special education is laid down in the Special Education Interim Act (ISOVSO, 1982) [62]. With this Act, special needs were first recognised and addressed within the Dutch education system. It also acknowledges the importance of the role of the parents by implementing pathways for the parents to partake in the education of their child, while simultaneously being educated themselves on their child’s condition (Articles 41a(4), 44 and 48). The role of the parents is mainly supportive, since the way of teaching is structured in the Act. Consequently, primary education was split between mainstream education and special education, with both being regulated by the government. The separation facilitated the inclusion of all children ages 4 to 12 in the education system. Amendments to the Secondary Education Act in 1998 resulted in the coverage of secondary special education for children with learning and behavioural difficulties and for students with moderate learning difficulties [63]. The educational trajectory for all children aged 4 to 18 was henceforth determined by three legislative acts, namely the Primary Education Act for mainstream primary education, the Secondary Education Act for secondary mainstream and special education and the Expertise Centers Act (previously ISOVSO) for special primary education. In an attempt to integrate special education into mainstream education, major reforms were made to these three Acts in 2012 in the Appropriate Education Act [64]. The reforms were aimed at broadening the scope of provision of services from care-based to support-based. This implied that the provision of special needs services was no longer restricted to medical needs. Instead, it could now address the full range of special education needs that limited children from fully participating in the education system. Nevertheless, there are still major barriers to the implementation of inclusive education.

A notable characteristic of the education system in the Netherlands is the freedom for any person to start a school and, by extension, organise its teaching, and determine the ideological, educational and religious principles on which the teaching is based. This freedom was included in the amendments to the Dutch Constitution in 1917 [65]. As a result, the Organisation for Economic Co-operation and Development (OECD) reports a high number of schools (especially primary schools) with a subsequent high diversity in educational perspective [66]. In order to determine the appropriate governmental funds for such a high number of different schools, the OECD reports that the Netherlands has adopted a scheme where the funds that a school receives is based on the number of students enrolled in that institution [66]. However, when relating back to the provision of SEN support in schools, this disadvantages schools smaller in size, as they receive less funding out of this scheme, while the costs of the SEN support provision remain the same.

Ultimately, SEN education in the Netherlands is fully integrated in national legislation and there is no specific action plan or strategy with regards to autism. Also, the universal right to education was addressed implemented well before the UDHR was established and adopted. The funding scheme can be considered a weakness that can form a barrier in the provision of SEN support, which applies mostly for smaller schools. Parental involvement is largely expressed through the freedom of choice of schools, especially primary schools. The law states schools should involve parents in the educational trajectory of children with SEN, yet there are no concrete guidelines in this regard. Schools can decide this for themselves.

### Germany

The right to equal treatment of people with disability is highlighted in Article 3 of the Basic Law (1949) [67], which was signed a year after the implementation of the UDHR. It also lays down the foundations for a system for special education in Article 7, while declaring in the same Article that the regulation of the education system is a responsibility of the Länder. At this point, the Basic Law only applied to the Federal Republic of Germany. The Basic Law became binding for Länder within the previous German Democratic Republic after the adoption of the Unification Treaty in 1990 [68]. In addition to the Basic Law on national level, each Land has its own constitution as well. For example, when Bavaria adopted its constitution in 1946 [69], it already included the right to education for all (Article 129). In North Rhein Westphalia, where the constitution was adopted in 1950, it states that every child has the right to education (Article 8), as well as that parents have the right to determine the education of their children, which forms the foundation of the education and school system (Article 10). The constitution of Lower Saxony was made to closely resemble the Basic Law in 1951 [70]. Because of its close resemblance, it avoided repetition of, for example, civil rights that were already mentioned in the Basic Law. Therefore, the right to education is not included in this document. Finally, since Saxony was part of Eastern Germany, which fell under the Soviet regime, it did not have a constitution comparable to the one in Western Germany until the first version was implemented in 1992 [71].

The education systems for the Länder are specifically laid down in their respective Education Acts (Bavaria, Article 19 [72]; North Rhein Westphalia, Article 20 [73]; Saxony, Article 13 [74]; Lower Saxony, Article 14 [75]). While each Act acknowledges the existence of SEN schools and their importance, there are slight differences in the regulation on how these schools should be implemented. Bavaria (Article 2), North Rhein Westphalia (Article 20) and Lower Saxony (Article 4) aim at including as many children in mainstream education and providing SEN services there, while still retaining specific SEN schools for children that are unable to attend mainstream education due to their disability. In contrast, the system in Saxony remains strongly split between mainstream and special education with the only overlap being the degrees that can be acquired. There are also other subtle differences between the Education Acts. Firstly, the Education Act of Bavaria lays down the conditions for a child to be eligible for admission into a SEN school in Article 19, stating that access is warranted when either a child cannot be supported or insufficiently supported and taught in mainstream education. Next, Article 30b discusses the aim to include as many children in mainstream education as possible, providing special education services where applicable and necessary. It also emphasises the right of parents to choose the type of school for the child in Article 44. Secondly, the Act of North Rhein Westphalia provides a specific definition of SEN, the general conditions for which SEN services are provided and the role of the educational institution towards the parents in Article 19. Article 20 specifies that the parents can deviate from the SEN service provision in mainstream schools and send the child to a special school. Thirdly, the Education Act of Saxony incorporates counselling centers in SEN schools in Article 13, whose responsibility is early detection and facilitate early interventions for children with disabilities, as well as offer disability-specific counselling for parents and teachers. Notably, active parental involvement is not included in this Education Act. Finally, the Education Act of Lower Saxony is similar to the Acts of Bavaria and North Rhein Westphalia, except that it provides an in-depth description on the establishment of inclusive education (Article 183c).

Even though the competence of education lies with the Länder, the Standing Conference of the Ministers of Education and Cultural Affairs (SCMECA) harmonised the development and organisation of special education by adopting several resolutions, most notably the Recommendations on the Organisation of Special Schools in 1972 [76]. Book Eight of the Social Code (1990) implemented youth services to assist parents in the development of the child and to support the child in his or her development and education [77]. It also identifies a wide range of supporting services in Articles 27 through 40 that can be used to provide assistance, based on the condition of the child with special needs and the environment it lives in. In Chapter 3 of the Recommendations on Special Needs Education in the Schools of the Federal Republic of Germany (1994) [78], developments were formulated that aimed to dismantle barriers and promote the equal participation of young people with disabilities in mainstream education and special education. Additionally, schools were given part of the responsibility to involve and educate parents with regards to the condition of their child and how to address this condition adequately outside of school. It also addresses the crucial role of the parents in helping a child establish sustained relationships with others over time. The SCMECA published recommendations on the education of children with autism in 2000 [79]. This document addresses the diagnostic criteria to be used for autism in schools in Chapter 3, the goals for the education system in general in Chapter 4 and the key points for every educational institution separately in Chapter 5. Book Nine of the Social Code on the rehabilitation and participation of people with disabilities (2001) specify in Articles 46 and 79 that early medical screening intervention is paramount in addressing disabilities as adequately as possible [80]. The term ‘medical screening’ does include non-medical social-paediatric, psychological, curative, psychosocial services and counselling of guardians using interdisciplinary services and facilities. Book Twelve of the Social Code, adopted in 2003, adds that the special circumstances in the family of the beneficiaries should be taken into account in the case of social assistance benefits [81]. Social assistance should encourage the family to help themselves and consolidate the cohesion of the family. In 2008, the SCMECA decided to amend the Recommendations on Special Needs Education in the Schools of the Federal Republic of Germany in order to respect the intentions of the CRPD in the Länder. The SCMECA adopted the Decision on Inclusive Education of Children and Young People with Disabilities in schools in 2011 [82]. The aim was to enable children and young people to be educated and trained together in mainstream education and to guarantee and develop the standards achieved in special education teaching, advisory, and support services.

Even though German SEN policy is harmonised, it still gives sufficient room for interpretation for the Länder, resulting in subtle differences. Saxony is notably less developed in terms of SEN policy, possibly because of the separation between Western and Eastern Germany after the Second World War and consequently differential development of their respective educational systems. With the Basic Law of the Federal Republic of Germany becoming binding for all of Germany since 1990, former Eastern German States were incentivised to develop similarly to Western Germany. Additionally, the provision of support and education for parents on the SEN or disability of the child is regulated through the harmonised law, yet the active involvement of parents in education is generally limited to the decision on school choice.

### Belgium

The right to education for all has been included in the Belgian constitution since 1831 (Article 24) [83, 84]. Over time, the regulation of education has been an area of tension in Belgium, as the government, linguistic communities and the church struggled for power. This struggle ended with the adoption of the School Pact in 1958 [83]. In the School Pact, a division was made between state-regulated schools and schools that were led by non-governmental institutions. Consequently, the School Act, adopted in 1959, laid down the foundation of all primary and secondary education systems in Belgium, including special education [85]. The need for special education for people with physical and mental disabilities started developing once the right to education for everyone was implemented [83]. This resulted in the adoption of the Act on Special and Inclusive Education in 1970 [86], which laid down the conditions under which children would be eligible to attend special schools, as well as the organisation of various levels of education. The amendments to the constitution of Belgium in 1988 resulted in the delegation of the competence of education to the individual regions in Belgium (Flanders, Wallonia and the German-speaking community) [22]. The right to education remained emphasised in Article 24. However, the article was expanded with the notion that parents have the ability to decide on the education of their child in order to guarantee his or her development [84]. Even though Belgium recognises a fourth legislative region (the Brussels-Capital region), it does not formulate its own legislation regarding education. Instead, it is a combination of the Flemish and the Wallonian system, depending on the core language of the school (e.g. Dutch-speaking schools fall under the Flemish system, while French-speaking schools belong to the Wallonian system) [87].

#### Flanders

The Decree on Primary Education, signed in 1997, built upon the foundation laid down by the Act on Special and Inclusive Education and states in Article 8 that mainstream education is responsible to educate all students between the ages of 6 and 12 and that schools can opt into a systematic and transparent cooperation with parents in case of additional needs [88]. Article 9 then explains that special education is provided to children whose development cannot be guaranteed by mainstream education, with Article 10 differentiating the several types of special education, taking the needs of the child into account (e.g. whether the child has an intellectual disability, impairment in motor skills, or autism). The CRPD is directly referenced in national law in the Decree on Equality, signed in 2008. The Decree states that any form of discrimination in the sector of education is prohibited in Article 20 [89]. Special education services in secondary education were implemented in 2010 through the ratification of the Codex on Secondary Education [90]. Article 357 specifies that SEN support can be provided for children that are able to partake in mainstream education [90]. Furthermore, parents are involved in the expansion of care based on SEN of the child, according to Article 3(44). The involvement includes both the care aimed from the parents to the child as well as the assistance a school can offer the parents. With the adoption of the Decree for Scholars with Special Education Needs was adopted in 2014 [91], measures were taken to create a more inclusive environment in primary and secondary education. The measures aimed to allow children with SEN to participate fully, effectively and on equal terms in mainstream education. Finally, a strategic plan for people with autism was passed by the Flemish Parliament in 2017 [92]. This strategic plan was developed in close relation with parents, using their insight to formulate the aims and goals of the plan. It aims to create and increase the opportunities for people with autism to actively participate in society and to increase their quality of life through the establishment of four principles: (1) the establishment of actions designed to be executed by people with and without autism; (2) participation and inclusion of people with autism in general society; (3) shared engagement between stakeholders; (4) and the investment on a select few interventions that have a clear focus and appropriate scale.

Ultimately, SEN service provision and right to education are repeatedly addressed in education policy. While a division between mainstream and special schools remain, actions have been put forward to develop a more inclusive school system, where segregation is less pronounced. Also, with the adoption of the autism-specific strategy, the needs of people with autism are recognised with an aim towards improvement. However, legislation has not incorporated a clear definition of the responsibilities for the diagnostic process of SEN. It only implies (rather than specifies) that mainstream schools are responsible for this, since this is the place where children can opt into additional support as well. Furthermore, mainstream education encourages parents to engage in the development of the child by providing education on SEN as well as counselling services.

#### Wallonia

The Act on Special and Inclusive Education was followed by the Decree on Primary Education in 2004 [93], which regulates and implements special primary and secondary education specifically for children with SEN. It states in Articles 25 (primary education) and 65 (secondary education) that a child can switch from a SEN school to a mainstream school based on the decision of the parents, given that the SEN school approves of this decision. The necessity of consent from the parents in the integration of the child with SEN into regular education is expressed in Article 134. Much like the Decree on Equality in Flanders, the Decree on Combatting Certain Forms of Discrimination, signed in 2008, specifies that discrimination on grounds of disability in the sector of education is strictly prohibited [94]. In doing so, it implements the aim of the CRPD in Wallonian law, although the CRPD is never directly referenced in the Decree. In 2014, the Decree on the Inclusion of People with Disability was ratified, which directly references the CRPD [95]. It specifies the responsibilities of support services towards children with disabilities in educational and extracurricular settings in Article 42. Article 43 specifies the scope of the support services by formulating four broad target groups. Children who belong to those groups are considered eligible for the support services specified by this Decree. Finally, the Decree on Inclusive Education for Social Promotion was signed in 2016 with the aim to include more children with SEN in mainstream education [96]. It acknowledges in Article 7 that SEN support can be materialistic, pedagogic or organisational, as well as specifies that inclusive education is best achieved by working towards the developmental goals that are set for the children with disabilities, rather than question those aims.

In short, while the education system in Wallonia started with a strict division between mainstream and special schools, recent legislation has aimed at bridging these two in order to create an environment of inclusive education. The right to education is notably less stipulated than in Flanders. Nevertheless, it is repeatedly reinforced by the adopted legislation on SEN. Additionally, parental involvement is limited, as it is only incorporated in the school choice of whether a child should switch from one education form to another, yet the latter still requires consent from the school that is currently attended. Furthermore, unlike the case in Flanders, there is no strategy in place that specifically focuses on the improvement of the environment for people with autism.

#### German-speaking Community

In 1990, a social service for people with disabilities was implemented through the Decree on the Installment of a Department of the German-Speaking Community for Persons with Disabilities [97]. This decree states in Article 4(4) that the aim was to offer early support for children with disabilities and their families, along with supporting the uptake in social systems, like the education system. However, due to the wide scope of this decree, it did not elaborate on specific parts of education (e.g. special needs education). A decree that regulates the responsibilities of staff in mainstream education was signed in 1998 [98], although specific parts of the decree are reported in Article 1 to be applicable to special education as well. In Article 23, it is stated that parents that raise the child can decide on the education trajectory of the child (being mainstream education, special education, or home-schooling). Additionally, Article 24 specifies that special needs support may be provided through a collaboration of multiple institutions with the aim to coordinate and complement the education provided. This was followed by the Decree on the Establishment of a Center for Education of Children with Special Needs in order to Improve Education for Children with Special Needs in 2009 [99]. The Center for Education for Children with Special Needs is introduced and regulated in Article 5. Article 6 specifies that the responsibilities include the provision of support to children with SEN in primary and secondary education, as well as to provide assistance to mainstream schools in order to improve the quality and inclusivity of education for children with SEN. Furthermore, Article 16 amends the aforementioned Decree of 1998, installing the aim of education for children with SEN, which is to enable them to live an independent and social life. On top of that, it formulates a clear definition of children with SEN and specified the responsibilities of school staff towards the parents that raise the child. Article 17 then amends the Decree on Primary Education to facilitate better distribution of resources based on the distribution of children with SEN. Following the aims set in the CRPD, the Decree on Combatting Certain Types of Discrimination was implemented in 2012 [100]. In Articles 3 and 4, it is stated that any form of discrimination on the grounds of disability is strictly prohibited in the education sector. Finally, the Decree on the Instalment of a Department of the German-Speaking Community for Self-Determined Life was ratified in 2016 [101]. It refers directly to the CRPD and replaced the Department of the German-Speaking Community for Persons with Disabilities installed by the decree in 1990. With the instalment of this department, the scope was increased significantly compared to its previous iteration, as is specified in Articles 6 and 11. More specifically, Article 6 states that the responsibilities towards the general public include creating awareness for SEN and its current support provision, as well as to conduct research to improve these services moving forward. Article 11 proceeds to specify that the responsibilities of the department towards children with SEN focus on providing guidance and support in their development, education and social integration.

In summary, the provision of SEN services is largely disconnected from the education system. Since the division of Belgium in three legislative areas, a separate institution has been put in charge of this provision in the German-speaking community. This separation of SEN service provision is reinforced repeatedly by newly adopted legislation, with the only exception being the adoption of the decree in 2009, which had as an aim to facilitate a better environment for inclusion in education for children with SEN. This creates a situation unique to the German community in Belgium, where schools are not in charge of the provision of SEN services, yet still have to facilitate a learning environment to incorporate them. Moreover, parental involvement has received little attention in the adopted legislation. The only statement of active parental involvement was the right to decide in which school a child will be enrolled. However, parents are taken into consideration when it comes to the implications of raising a child with SEN, as the independent institutions that provide SEN also have the responsibility to provide information and guidance to parents. Finally, the general public is put in a position to be more cognisant of the impact of SEN on children and their families when compared to Flanders and Wallonia, because recent policy adoptions have included measures to raise awareness on SEN and its implications.

## Discussion

This study aimed to map relevant autism and SEN policies in the Netherlands, Germany and Belgium and to investigate to what extent family was involved in the provision of special needs and the creation of SEN policy on autism nationally and internationally. In doing so, we found that all regions under study have taken significant action to empower children with SEN in their development. Additionally, the critical role of parents in the development of children with SEN is acknowledged by the UN and all Member States under study. Interestingly, there is very little mention of parental involvement in EU legislation.

Firstly, the UDHR can be considered the critical juncture that led to the current state of universal right to education internationally and in the three countries under study. Since the adoption of the UDHR, legislative documents have referred to and reinforced the right to education for everyone nationally and internationally by implementing measures to allow children from all backgrounds and with any condition or disability to access the education system. More specifically, the implementation of the universal right to education started with the adoption of the Special Education Interim Act in the Netherlands, the consecutive measures to harmonise special education across Germany in order to make education more accessible for children with SEN and the ratification of the Act on Special and Inclusive Education in Belgium respectively. After several decades of implementing and regulating access to education for everyone nationally, the CRPD was adopted and proceeded to emphasise children with SEN as core recipients of the universal right to education. As a response, the countries under study aimed to adopt a system of inclusive education, where children with and without SEN could participate in education together. In hindsight, the adoption of the UDHR and CRPD has set the pathway for the current environment of the right to education. In terms of family involvement, it is interesting to note that UN policy recognises the importance of family in the development of children with disabilities, yet it remains untranslated in the EU initiatives. Regardless, the countries under study do mention parents in their policy to various degrees, which will be discussed later.

Secondly, legislation that focuses specifically on SEN or developmental conditions acknowledges the barriers to implementing inclusivity in mainstream education and aims to target these barriers by implementing respective measures, such as assisting mainstream schools in the provision of SEN services and providing education for teachers and parents on the implications of having SEN as a child.

Thirdly, the responsibilities of providing SEN services are almost unanimously within the school system, with the only exception being the German-speaking community in Belgium. Consequently, financing schemes for schools have become more relevant for the provision of SEN systems. Taking the Netherlands as an example, schools receive funding based on a capitation formula of the number of children that attends a school, while the costs of providing SEN services remain the same. Consequently, schools that have a lower number of attending children are disadvantaged due to receiving less funding, while it is expected of them to offer similar quality of education to all attending children. For them, the financing mechanism forms a significant barrier to providing SEN services. On the other hand, schools that have a high number of attendees will have a comparatively easier time financing additional services for children with SEN.

Fourthly, family involvement in mainstream education is generally limited to the parents or guardians of the child, who receive the right to choose the type of school that their child attends. In the legislation on special needs education, the education of parents has been acknowledged as an important element and is often facilitated by the institution that is responsible for the provision of SEN services. Furthermore, parents in the Netherlands and Flanders are included in the provision of SEN services, taking on a supportive role, while the other countries or regional governments do not include them in the provision of SEN services, but only educate them on how to address the SEN outside the school environment. Finally, the introduction of the General Data Protection Regulation has formed new barriers to take into account when implementing SEN services, which can hinder the access to education for children with SEN.

There are some contrasts that need to be addressed as well. Firstly, the definition of SEN that is implemented in the legislation of some countries under study does not refer to a common consensus of a definition for SEN. Therefore, it is possible that a heterogenous cross-country environment is created where one country may identify SEN different from another. Secondly, the responsibility for policy making in education lies on different levels in the countries under study. The Netherlands decides and implements nationally, Germany decides nationally, but the Länder have to implement in regionally, and Belgium decides and implements regionally. The difference in these approaches is that regions that are relatively small (like the German-speaking community in Belgium) or come from a different legislative background (like Saxony in Germany) are put in a position where priorities need to be considered when allocating time and resources to a relatively small group of people. When looking at the German-speaking community, specifically, it becomes apparent that every adoption of new legislation is preceded by an adoption of binding legislation by the UN or EU.

When examining the influence of the UDHR more closely, it becomes apparent that the values of the UDHR have been integrated in every constitution under study and have been repeatedly referred to and reinforced by the subsequent policy adoptions, especially policies that address right to education and empowerment of people with disabilities. The Charter on the Fundamental Rights of the European Union, upon adoption, became binding in its entirety for all EU Member States, ensuring the adoption of the legislation pooled into this document at national level. Furthermore, the CRPD has been widely referenced and implemented in education policy of Member States. The Salamanca Statement, however, has taken nearly a decade to catch on at the national level, as countries were still adapting to the implementation of SEN support in general. Therefore, progression towards inclusive education has been delayed until the services were properly implemented. Ultimately, the implementation of the aims of the Salamanca Statement has been lacklustre, while the UDHR and the CRPD have been integrated significantly better. Possible reasons for the diminished implementation of the Salamanca Statement include the variance in education systems and the lack awareness of the benefits of inclusive education to people with SEN. When aligning the outcomes of this paper with the outcomes of the work by Roleska and Roman-Urrestarazu and colleagues [29], it becomes apparent that all countries included thus far in the mapping project have attempted to create an environment of inclusive education. However, in doing so, they try to fuse two systems that differ significantly in scale and regulation. Hence, the feasibility of an EU guideline that addresses the common aspects of mainstream and special education when attempting to implement inclusive education should be looked into.

While mapping relevant policies with regards to SEN internationally and in countries under study, it became apparent that the values and goals of the UDHR and CRPD have been translated into national policy and are being reinforced in the most recent policy initiatives as well. The attempts at implementing inclusive education further is a prime example of this, as it further aims to realise horizontal equity and facilitate access to education and growth for all children. Also, the role of family involvement in the educational trajectory of children with SEN in each country has been reported, which came down to the involvement of parents and guardians. More specifically, there is some guidance on international level on the involvement of parents. Each country under study involves parents in start of the education process by allowing them to choose the school. Additionally, parents with children of SEN in Germany and Flanders actively receive guidance on how to address the SEN of their child at home. Other countries have not implemented such measures for parents.

However, this study did not come without its limitations. Firstly, the scope of the study only included three counties, and therefore, the results cannot be generalised to countries not included in this analysis. It is also difficult to produce clear conclusions on the average level of the fulfilment of the right to education considering the qualitative nature of this work. Secondly, non-governmental organisations were not included in this study, unless their work was laid down in legislation in some way, like the Charter for Persons with Autism by Autism-Europe. Finally, the scope of this study only included children with autism. As a result, adults with autism that are still in an educational environment are not included in this analysis, while they may experience learning difficulties all the same.

This study also provides some opportunities for further research. Firstly, mapping the autism SEN policy relevant to adult education would extend the approach to understand how this population group’s needs can be met better. Secondly, the prevalence of autism and SEN in children in Germany should be mapped. Thirdly, while an overview of policy is provided in this research, adherence to and implementation of policy was not addressed. Therefore, a survey-based study on adherence to SEN policy in autism may contribute valuable insight to improving policymaking, so barriers can be accounted for moving forward. Fourthly, aside from education, employment is an area that can also be heavily affected by SEN and autism. A policy analysis in the employment sector and how SEN in autism are accounted for in this sector can contribute to the process of improving the employment possibilities of people with autism. Fifthly, the cognitive and social impacts of inclusive education in autism have been extensively investigated. However, it is not reported whether those results are universal for children with autism and SEN or whether that is offset by a degree of learning difficulties among children with autism. This is especially important considering the high proportion of people with autism with intellectual disabilities. Investigating how learning disabilities with autism and inclusive education can aid in tailoring better fitted education for children with SEN. Sixthly, one of the most common problems that inclusive education encounters is that a teacher cannot address the difficulties of a child with SEN, because there are too many other children to address as well. Therefore, a longitudinal evaluation that assesses the outcomes of children with and without SEN in inclusive education when assigned to smaller classes could provide major insight in determining the feasibility of integrating inclusive education in the education system currently in place. Finally, the effects of the General Data Protection Regulation have the potential to significantly complicate collaborations between institutions that deal with SEN data. A proper study to map these difficulties and address solutions should be conducted.

Finally, there are two major policy recommendations that can be extrapolated from these findings. Firstly, the current trend of inclusive education is to systematically introduce children with SEN into mainstream schools without changing the structure of the mainstream education. However, the structure of that system is a leading factor in why children with SEN were not able to participate fully. While additional services are made available, it may be more feasible to push incremental changes (e.g. smaller class sizes, individual learning trajectories) to the education system to alleviate stress from teachers, who can then attend to the needs of the children properly, thus increasing the quality of assistance that is advocated for in both international and national policy. Secondly, our findings indicate that some countries still operate under different definitions of SEN. Therefore, we recommend official guidelines on the definition and impacts of groups of SEN to be drafted at an international level. The report by Carroll and colleagues [10] already makes a division between several types of SEN and this could be used as underlying framework. As a result, it could benefit the transferability of SEN policy, since it removes the potential limiting factor of differing terminologies across countries.

## Conclusion

This study provided information on the right to education of people with autism in the Netherlands, Germany and Belgium. In all three countries, the values of the UDHR on the right to education have been integrated in national legislation. Appropriate SEN services and special schools are in place so that all children can enjoy their right to education. All countries regulated the provision of SEN through schools, except for the German-speaking community in Belgium, which implemented a separate department for this. Active parental involvement is included in the legislation of the Netherlands and Flanders, while the other regions involve them through providing education for the SEN of the child and by leaving them the decision which school the child will attend. To this day, research on education and autism policies is limited, both in the EU and globally and is an important gap in autism research.

## Supplementary information

**Additional file 1.** An overview of the demographics of the countries under study. Description: BA = Bavaria; NRW = North Rhein Westphalia; S = Saxony; LS = Lower Saxony; FL = Flanders; WA = Wallonia; GC = The German Speaking Community in Belgium. * Dutch population size was found using data from Eurostat [32], the size of the Länder was reported by the German Statistics Office [34], and the Belgian population size was reported by the Belgian Federal Government [33]. ** Autism prevalence rates in the Netherlands were reported by Roelfsema and colleagues [20], in Germany by Bachmann and colleagues [35], and in Belgium by Dereu and colleagues [36].

**Additional file 2.** An overview of the countries under study with regards to demographics, autism prevalence, and SEN policy. Description: BA = Bavaria; NRW = North Rhein Westphalia; S = Saxony; LS = Lower Saxony; FL = Flanders; WA = Wallonia; GC = The German Speaking Community in Belgium. * Dutch population size was found using data from Eurostat [32], the size of the Länder was reported by the German Statistics Office [34], and the Belgian population size was reported by the Belgian Federal Government [33]. ** Autism prevalence rates in the Netherlands were reported by Roelfsema and colleagues [20], in Germany by Bachmann and colleagues [35], and in Belgium by Dereu and colleagues [36].

**Additional file 3.** An overview of the specific policies and impacts of the countries under study. Description: This overview provides a comprehensive version of the results section.

## Data Availability

While all data are publicly available, a list of used documents along with their source has been included.

## Abbreviations

ASCs: Autism spectrum conditions—also referred to as autism
EU: European Union
SEN: Special education needs
EDUCAUS: European Consortium for Autism Researchers in Education
UDHR: Universal Declaration of Human Rights
CRPD: Convention on the Rights of Persons with Disabilities
UNESCO: United Nations Educational, Scientific and Cultural Organisation
ISOVSO: Special Education Interim Act
OECD: Organisation for Economic Co-operation and Development
SCMECA: Standing Conference of the Ministers of Education and Cultural Affairs
