# Supplementary material for: Autism and family involvement in the right to education in the EU: policy mapping in the Netherlands, Belgium and Germany
Source: Mol Autism. 2019 Dec 9;10:43. doi: 10.1186/s13229-019-0297-x (PMC6902602; doi:10.1186/s13229-019-0297-x)
Supplement: Supplementary file 1 — Additional file 1. An overview of the demographics of the countries under study. Description: BA = Bavaria; NRW = North Rhein Westphalia; S = Saxony; LS = Lower Saxony; FL = Flanders; WA = Wallonia; GC = The German Speaking Community in Belgium. * Dutch population size was found using data from Eurostat [32], the size of the Länder was reported by the German Statistics Office [34], and the Belgian population size was reported by the Belgian Federal Government [33]. ** Autism prevalence rates in the Netherlands were reported by Roelfsema and colleagues [20], in Germany by Bachmann and colleagues [35], and in Belgium by Dereu and colleagues [36]. [file 13229_2019_297_MOESM1_ESM.docx]

|  | The Netherlands | Germany | | | | Belgium | | |
| --- | --- | --- | --- | --- | --- | --- | --- | --- |
|  |  | BA | NRW | S | LS | FL | WA | GC |
| Population size | 17.1 mln* | 12.9 mln* | 17.8 mln* | 4 mln* | 7.9 mln* | 6.4 mln* | 3.6 mln* | 75.2 thousand* |
| Autism prevalence (%) | 0.6-2.3** | German total: 0.4** | | | | Belgian total: 0.6** | | |
