# Supplementary material for: Autism and family involvement in the right to education in the EU: policy mapping in the Netherlands, Belgium and Germany
Source: Mol Autism. 2019 Dec 9;10:43. doi: 10.1186/s13229-019-0297-x (PMC6902602; doi:10.1186/s13229-019-0297-x)
Supplement: Supplementary file 2 — Additional file 2. An overview of the countries under study with regards to demographics, autism prevalence, and SEN policy. Description: BA = Bavaria; NRW = North Rhein Westphalia; S = Saxony; LS = Lower Saxony; FL = Flanders; WA = Wallonia; GC = The German Speaking Community in Belgium. * Dutch population size was found using data from Eurostat [32], the size of the Länder was reported by the German Statistics Office [34], and the Belgian population size was reported by the Belgian Federal Government [33]. ** Autism prevalence rates in the Netherlands were reported by Roelfsema and colleagues [20], in Germany by Bachmann and colleagues [35], and in Belgium by Dereu and colleagues [36]. [file 13229_2019_297_MOESM2_ESM.docx]

|  | The Netherlands | Germany | | | | Belgium | | |
| --- | --- | --- | --- | --- | --- | --- | --- | --- |
|  |  | BA | NRW | S | LS | FL | WA | GC |
| Population size | 17.1 mln* | 12.9 mln* | 17.8 mln* | 4 mln* | 7.9 mln* | 6.4 mln* | 3.6 mln* | 75.2 thousand* |
| Autism prevalence (%) | 0.6-2.3** | German total: 0.4** | | | | Belgian total: 0.6** | | |
| Type of education | Mainstream, mixed, special | Mixed, special | Mixed, special | Mainstream, special | Mixed, special | Mainstream, mixed, special | Mainstream, mixed, special | Mainstream, mixed, special |
| Education funding scheme | Based on number of students | Defined in respective Education Act | | | | National subsidies for schools in all legislative regions | | |
| Level of education policy | National | Harmonised nationally | | | | Regional | | |
| Family involvement in education | School choice | School choice, support and social assistance for parents | | | | School choice, parents encouraged to engage in education | School choice | School choice |
| Right to education | Constitution, Education Acts | The Basic Law, individual Constitutions, Book Twelve of the Social Code | | | | Belgian Constitution, School Act, Decree on Primary Education, Codex Secondary Education | Belgian Constitution, Decree on Primary Education, Decree on Combatting Certain Forms of Discrimination | Belgian Constitution, Decree on the Instalment of a Department o for Persons with Disabilities, Decree on Combatting Certain Types of Discrimination |
| Autism policy | Not specified | Recommendations on the Education of Children with Autism | | | | Strategic Plan for Autistic People | Not specified | Not specified |
| SEN policy | Special Education Interim Act, Appropriate Education Act | Recommendations on the Organisation Special Schools, on the Special Needs Education and its amendments, Decision on Inclusive Education, Book Eight of the Social Code, | | | | Decree on Primary Education, Codex Secondary Education, Decree for Scholars with Special Education Needs | Decree on Primary Education, Decree on the Inclusion of People with Disability, Decree on Inclusive Education for Social Promotion | Decree on Responsibilities of Education Staff, Decree on the Establishment of a Center for Education of Children with Special Needs, Decree on the Instalment of a Department for Self-Determined Life |
| SEN service provision | Schools | Schools | | | | Schools | Schools | Independent department |
